# Investigation of Tensile Creep of a Normal Strength Overlay Concrete

**DOI:** 10.3390/ma11060993

**Published:** 2018-06-12

**Authors:** Martin Drexel, Yvonne Theiner, Günter Hofstetter

**Affiliations:** Unit for Strength of Materials and Structural Analysis, Institute of Basic Sciences in Engineering Sciences, Innsbruck University, Technikerstr. 13, A-6020 Innsbruck, Austria; Yvonne.Theiner@uibk.ac.at (Y.T.); Guenter.Hofstetter@uibk.ac.at (G.H.)

**Keywords:** concrete, tensile creep, shrinkage, mass water content

## Abstract

The present contribution deals with the experimental investigation of the time-dependent behavior of a typical overlay concrete subjected to tensile stresses. The latter develop in concrete overlays, which are placed on existing concrete structures as a strengthening measure, due to the shrinkage of the young overlay concrete, which is restrained by the substrate concrete. Since the tensile stresses are reduced by creep, creep in tension is investigated on sealed and unsealed specimens, loaded at different concrete ages. The creep tests as well as the companion shrinkage tests are performed in a climatic chamber at constant temperature and constant relative humidity. Since shrinkage depends on the change of moisture content, the evolution of the mass water content is determined at the center of each specimen by means of an electrolytic resistivity-based system. Together with the experimental results for compressive creep from a previous study, a consistent set of time-dependent material data, determined for the same composition of the concrete mixture and on identical specimens, is now available. It consists of the hygral and mechanical properties, creep and shrinkage strains for both sealed and drying conditions, the respective compliance functions, and the mass water contents in sealed and unsealed, loaded and load-free specimens.

## 1. Introduction

**Figure 1:**
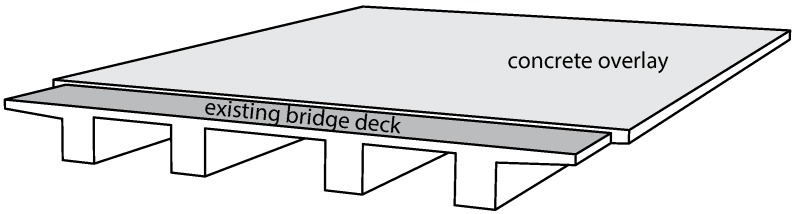
Schematic illustration of strengthening an existing concrete bridge deck by adding a concrete overlay.

Strengthening measures of existing concrete structures by adding concrete overlays (Figure 1) are becoming more and more important. The primary reasons are the age of existing concrete bridges [1,2], the fast increase of the traffic volume during the last decades, particularly with respect to heavy traffic [3,4], the increasing permissible gross vehicle weights [3,5] and the increasing numbers of authorized heavy goods transports [3,4,5].

**Figure 2:**
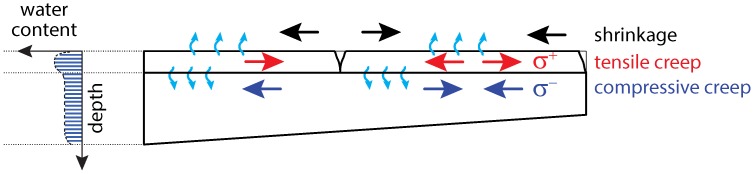
Schematic view of the moisture distribution through the depth of a strengthened structure together with the restraint effects due to drying and the related stresses.

The behavior of a concrete structure strengthened by an overlay is mainly characterized by overlay volume changes, related restraint effects in both the overlay and substrate concrete as well as the relaxation of the restraint stresses due to the viscoelastic material behavior of concrete. During the curing process, the early age overlay volume changes result from the exothermic process of cement hydration and related temperature changes. The macroscopic volume reduction of concrete caused by cement hydration after initial setting without moisture exchange between concrete and surrounding environment is referred to as autogenous shrinkage [6]. At later ages, when the curing process is completed, the overlay volume changes are mainly caused by a combination of autogenous shrinkage and drying shrinkage. The latter refers to the volume reduction due to loss of moisture to the environment [7]. The restraint of overlay shrinkage deformations by the substrate concrete causes tensile stresses in the overlay at an early age, shear stresses at the interface, and compressive stresses in the substrate concrete [8]. The tensile stresses in the overlay are most critical and may result in extensive cracking, if the tensile strength is exceeded [8,9] or they remain as residual stresses in the overlay [9]. In addition to cracking, debonding is another overlay failure mechanism leading to local delamination and spalling [8]. In an intact concrete overlay tensile creep strains counteract the shrinkage strains [9,10]. Furthermore, the compressive stresses in the adjacent substrate concrete result in compressive creep strains, and, hence, they further reduce tensile stresses in the overlay [8]. Restraint shrinkage of the overlay, resulting in tensile stresses, which may cause cracking, the simultaneously acting compressive stresses in the substrate concrete together with the moisture distribution in both the overlay and the substrate concrete are schematically depicted in Figure 2. For ensuring good bond properties between the substrate and the overlay concrete, according to the Austrian guideline RVS 15.02.34 [11] for the design and detailing of concrete overlays, the concrete surface of the existing structure must be prepared by high-pressure water jetting. Furthermore, for preventing early drying of the overlay, the substrate concrete must be wetted thoroughly before placing the concrete overlay.

It is generally accepted that creep of concrete is composed of basic and drying creep. Basic creep is defined as the creep of concrete occurring under sealed conditions, i.e., no moisture exchange with the environment, whereas drying creep is defined as the creep component additionally developing to basic creep in a loaded specimen exposed to drying [7]. Mechanisms of compressive creep of concrete under sealed and drying conditions have extensively been investigated for many decades. Hence, a large amount of test data is available in the open literature. For instance, a database comprising 1400 compressive creep tests was assembled under the guidance of Z. P. Bažant at Northwestern University [12]. Contrary to the large body of literature on compressive creep, the literature regarding creep tests in tension is rather scarce, amongst other reasons, because such tests are much more complicated to perform [13,14,15]. According to Ranaivomanana et al. [16,17], previous research on tensile creep has mainly been focused on the viscoelastic behavior of young concrete, i.e., when simultaneous physical and chemical changes of hardening concrete make the measurement and interpretation of creep in tension difficult [13].

The conclusions drawn from comparative investigations of compressive and tensile creep are not consistent [18,19,20]. For instance, several researchers reported that creep strains in tension are larger than the ones in compression [21,22,23,24,25]. On the contrary, other researchers presented either opposite results [16,17,18,26,27] or similar strain magnitudes for creep in tension and compression [19,20,26,28]. Moreover, the conclusions should be treated with caution, especially since the experimental procedures, like the sealing procedure, test conditions, load level and loading age, and the measurement systems used for recording the very small arising displacements, are quite different [16,27]. For instance, time-dependent deformations were detected by means of linear variable differential transformer (LVDT) transducers [16,18,19,20,26,27], embedded acoustic or vibrated-wire strain gauges [23,24], demountable mechanical strain gauges (e.g., Whittemore gauge) [21,22,25] or electrical resistance strain gauges (quarter-bridge [16,17] or full-bridge arrangement [28,29]). High demands are required for the measurement accuracy since the applied tensile stress is low with correspondingly small strains [22]. Regarding sealing procedures and test conditions, basic creep in tension was determined on specimens either sealed with several layers of adhesive aluminum foils [16,18,19,25,26,27,30] or kept wet through immersion in water [23,24] or by means of water jackets [22].

Several authors [23,25,31,32,33] have tried to explain the mechanisms of tensile creep in terms of a combination of the mechanisms involved in compressive creep, i.e., water seepage [34], viscous shear [35], and microcracking [36]. Bažant and Chern [37] introduced the concept of stress-induced shrinkage and concluded that some sort of both water seepage and viscous shear appear to be involved. This conclusion follows from the idea of microdiffusion of water between gel pores and adjacent capillary pores, and from the dependence of creep viscosity on the time rate of change of pore humidity [37]. Altoubat and Lange [38] developed a technique to separate the different mechanisms of tensile drying creep and showed that in plain concrete the Pickett effect has two sources, namely stress-induced shrinkage and microcracking. The latter was successfully monitored by means of the acoustic emission technique during creep tests in compression [18,39] and flexure [40].

Concerning the non-linearity of tensile creep, it is usually accepted that up to a certain stress/strength ratio the creep strains are proportional to the applied stress, and, above that limit the creep strains disproportionately increase as a result of progressing microcracking. The results of several researchers [26,32,41,42] indicate linear viscoelastic behavior in tension up to a stress/strength ratio of 60–78%, depending on the concrete composition and the test conditions, whereas in [22,33] the proportionality of stress and tensile creep strain was only observed up to a stress/strength ratio of 50%.

Although several researchers investigated the influence of the moisture content on the creep behavior of concrete, there is hardly any information in the literature about the evolution of the moisture content in sealed or unsealed loaded specimens. Wyrzykowski and Lura [43,44] provided test data on the evolution of internal relative humidity during basic creep tests in compression and short-term loading tests in both compression and tension. Thereby, the relative humidity was determined by means of miniature sensors, which were placed in precast holes at central parts of the specimens and the remaining openings were sealed with paraffin film.

In summary, in concrete overlays (i) tensile stresses due to restrained autogenous and drying shrinkage strains develop already at early ages; (ii) the evolution of drying shrinkage strains depends on the decrease of the water content in the overlay due to hydration and migration of moisture between the overlay and the substrate concrete and (iii) the tensile stresses are reduced by tensile creep. In order to capture the described phenomena in the present experimental study on small-scale specimens, the shrinkage and tensile creep behavior of overlay concrete is determined on sealed and unsealed specimens, exposed to drying and/or loaded at concrete ages of 2, 7, and 28 days. All specimens are equipped with multi-ring sensors, embedded in the cement paste matrix, for measuring the evolution of the mass water content. Hence, the impact of the concrete age at load application on the moisture content and the influence of the moisture exchange between specimens and ambient air of lower ambient humidity on the shrinkage and tensile creep behavior of overlay concrete are studied in detail.

The present study is connected to a recent study [45], in which the compressive creep behavior and the respective evolution of the moisture content have been investigated for the same concrete mixture. These results are available in the database assembled at Northwestern University [12]. The specimen shape and size, the age of concrete at loading, the sealing technique, the measurement device as well as the load level are the same as in the present study. Hence, any size effect due to different specimen sizes, as observed in [26], or any influence of different sealing techniques and measurement devices can be excluded.

## 2. Experimental Procedures

### 2.1. Material Characterization and Specimen Preparation

The composition of the investigated concrete mixture is given in Table 1. It has been used for the strengthening of an existing reinforced concrete bridge deck by adding a concrete overlay [46].

The air-entraining agent based on synthetic tensides was used to meet the high demands concerning the frost resistance of a concrete overlay (exposure class XF3 according to [47]). The lignosulfonate- based plasticizer was added to the concrete mixture for increasing the flowability of concrete to ensure good bond properties with the substrate concrete [47].

The mixing procedure was as follows: Coarse aggregates, sand, and cement were first mixed dry in a rotary pan mixer for a period of 1 minute. Then the solution of air-entraining agent and mixing water was gradually added during the next minute, followed by adding the plasticizer and continuing the mixing for another 3 min. The concrete age at *t* = 0 is referred to the time when cement and water come in contact during mixing. Fresh concrete properties were determined according to the ONR 23303 [48] and revealed a flow spread of 493 mm, a unit weight of 2320 kg/$m^{3}$, an air content of 6.0%, and a total water content of 7.2% of concrete mass.

Immediately after determining the properties of fresh concrete and a further short mixing, the following specimens were cast:

- Cubes with an edge length of 150 mm for determining the average cube compressive strength,
- Prisms with dimensions of product-units = single 100×100×400 mm for determining the average Young’s modulus,
- Prisms with dimensions of product-units = single 110×110×400 mm for determining the water desorption isotherm, recently published in [45],
- Cylinders with 150 mm diameter and 300 mm height for determining the average splitting tensile strength,
- Cylinders with 150 mm diameter and 450 mm height for determining both the average uniaxial tensile strength and compressive strength,
- Cylinders with 150 mm diameter and 450 mm height for the compressive creep tests, recently published in [45], the tensile creep tests, and the companion shrinkage tests.

All specimens were cast in steel molds, except for the cylindrical specimens of 450 mm height, which were cast in longitudinally slotted PVC molds. After casting, all specimens were covered with polyethylene sheets and stored in a climatic chamber at (20±1) ${}^{\circ }C$ and (65.0 ± 2.5)% relative humidity. They were demolded at the concrete age of 24 h. After demolding, all specimens were immediately sealed by one layer of cling film, covered by one layer of bitumen-coated aluminum foil, except for the cylindrical specimens, which were used for determining the uniaxial tensile strength and for investigating the tensile creep behavior. These specimens were first sealed by several layers of cling film and then, approximately 10 mm thick slices were removed at both ends using a disk saw. In order to obtain cuts perpendicular to the specimen axis, an apparatus similar to a roller type chassis dynamometer was used. The ends were cleaned with compressed air before being glued to 30 mm thick steel plates using the adhesive SikaDur^®^-31. About 7 h later, the specimens were additionally sealed by one layer of bitumen-coated aluminum foil. After sealing, all specimens were stored again in the climatic chamber.

The average cylinder compressive strength $f_{cm}$ and the average Young’s modulus $E_{cm}$ were determined according to the ONR 23303 [48], and the average splitting tensile strength $f_{ctm,sp}$ was determined according to the Austrian standard ÖNORM EN 12390-6 [49], however, all those specimens were sealed until testing. In order to classify the investigated overlay concrete according to its strength class, 3 cubes were cured for one week in water followed by storing the specimens in the climatic chamber at (20±1) ${}^{\circ }C$ and (65.0 ± 2.5)% relative humidity until testing at the concrete age of 28 days. According to the average cube compressive strength of 45.2 MPa, the concrete is classified as C30/37 [50]. Similar to the tests performed by Van Mier et al. [51], the uniaxial tension tests for determining the average uniaxial tensile strength $f_{ctm}$ were performed on cylindrical specimens (sealed until testing) with a circumferential notch of 5 mm width and 16 mm depth at half-height. The depth of the notch corresponds to half of the maximum aggregate size of 32 mm (cf. Table 1). The tests were conducted in a Shimadzu Autograph 100 kN universal testing machine at a constant displacement rate of 0.12 mm/min. Two identical loading fixtures were used containing a ball joint to ensure centric loading of the specimen. The steel plates at both ends of the notched specimens were connected to the fixtures by using a 18 mm pin. In Table 2, the mean mechanical properties of the investigated concrete and the corresponding standard deviation (in brackets) are given at the concrete ages of 2, 7, and 28 days. Each mean value was obtained from 3 specimens.

The normalized uniaxial tensile strength at concrete ages of 2 and 7 days amounts to about 65% and 94% of the respective strength at 28 days, whereas the respective normalized splitting tensile strength amounts to about 68% and 90% of the respective strength at 28 days. The respective normalized compressive strength amounts to about 51% and 81% of the respective strength at 28 days [45]. These findings are confirmed by the experiments of De Schutter and Taerwe [52]. Furthermore, the ratio between uniaxial tensile strength and splitting tensile strength at concrete ages of 2, 7, and 28 days amounts to 1.00, 1.09, and 1.05, respectively. The latter is in agreement with results reported by Kanstad et al. [53] and Malárics and Müller [54]. The Young’s modulus at concrete ages of 2 and 7 days normalized to the corresponding 28-day value amounts to 76% and 86% [45]. Hence, at early ages, the material stiffness evolves faster than the tensile strength or the compressive strength [52,55].

In addition, the water desorption isotherm, characterizing the relationship between pore relative humidity and mass water content at constant temperature, was determined on thin concrete slices for 5 different values of ambient relative humidity. The thin concrete slices were obtained from the prisms with dimensions of product-units = single 110×110×400 mm by wet sawing.

### 2.2. Test Program

Immediately after demolding, sealing, and installing displacement transducers, autogenous shrinkage was recorded for one specimen from the concrete age of 24 h.

Six specimens each were used for the investigation of basic creep and drying creep in tension and in compression. The specimens were loaded at concrete ages of 2, 7, and 28 days to the load level of 30% of the average uniaxial tensile strength or cylinder compressive strength [45], respectively, determined at the concrete age at loading. In addition, companion specimens were used for measuring combined autogenous and drying shrinkage. They were unsealed and the measurements were started at the same ages as for the loaded specimens.

Similarly to Wyrzykowski and Lura [44], the impact of tensile loading on the moisture content was examined by subjecting specimens to a short term cycle of unloading and reloading to the previously applied tensile stress. These unloading/reloading cycles were not applied to the specimens loaded at the age of 2 days because of the small magnitude of the applied tensile stress.

The tensile creep specimens were unloaded at the age of 216 days and the measurements were finished at the age of 232 days.

**Figure 3:**
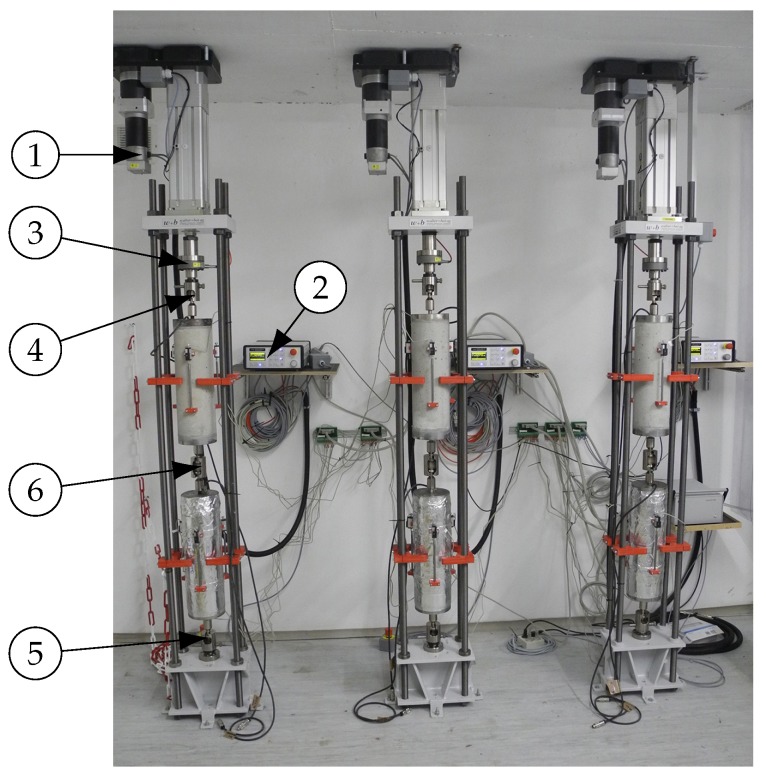
Experimental setup for determining tensile creep strains of sealed and unsealed specimens: ➀ linear actuator, ➁ digital controller, ➂ load cell, ➃ and ➄ spherical calottes, and ➅ two-sided spherical calotte.

At the respective concrete ages at loading, creep and companion shrinkage specimens were equipped with displacement transducers and the creep specimens were mounted on top of each other either in the hydro-pneumatic compressive creep devices (described in detail in [45]) or in the electromechanical tensile creep devices (walter+bai ag Testing Machines, shown in Figure 3).

After removing the sealing of the specimens for investigating combined autogenous and drying shrinkage and drying creep in tension and compression, the recording of measurement data was started and the creep specimens were loaded up to the prescribed load. The tensile load was applied at a constant loading rate of 1.0 kN s^−1^ by means of a linear actuator driven by a servo motor. Each tensile creep device was controlled via a digital controller. The actual tensile load applied to the specimens was measured with a load cell arranged between the linear actuator and the upper connection point of the specimens. Both the upper and lower connection points were designed as spherical calottes. Moreover, the specimens were connected to each other with an adapter, which was designed as a two-sided spherical calotte. Hence, proper alignment of the central axes of the specimens is ensured and possible bending effects in the specimens due to eccentric tensile load application were minimized.

The longitudinal deformations of both creep and companion shrinkage specimens were measured by means of 3 displacement transducers fixed along the perimeter of each specimen at distances of 120${}^{\circ }$. The total strain of each specimen was calculated as the average ratio of the 3 measured length changes over the measurement length of 200 mm. The arrangement of the displacement transducers allowed verifying whether the creep specimens were loaded uniformly. For both loading in tension and compression, at the end of load application, only a small variation of the instantaneous deformation at the 3 measuring points was noticed. Moreover, during load application, the longitudinal deformations were recorded with a measuring rate of 5 Hz, and then recorded at intervals of 10 to 20 min.

**Figure 4:**
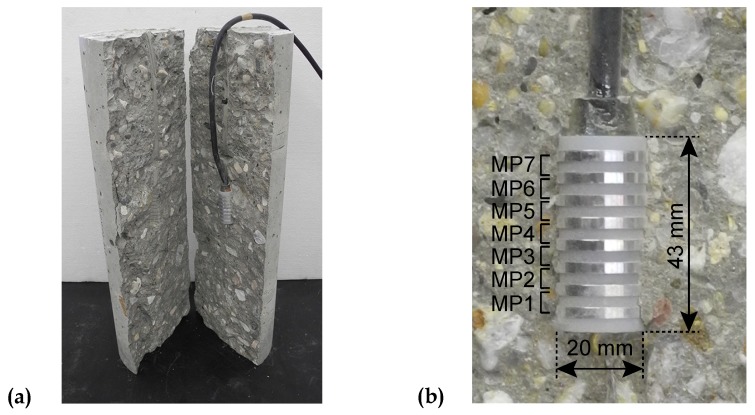
(**a**) Section through the concrete specimen with embedded multi-ring-sensor (MRS) and (**b**) MRS with the respective measuring points (MP) and dimensions.

At the center of each cylindrical specimen (Figure 4a), the evolution of the mass water content was determined by means of a multi-ring-sensor (MRS), which was developed at the Institute for Building Materials Research of the RWTH Aachen University (ibac) [56,57,58]. The sensor used in this study consists of 8 stainless steel rings, separated by insulating polymer rings. Its dimensions are similar to the maximum grain size of 16/32 mm. The electrolytic resistance is determined between each pair of adjoining stainless steel rings by impedance measurements [57]. The 7 measuring points (MP) are shown in Figure 4b. The respective mass water contents were determined by means of a calibration curve for hardened concrete. The latter characterizes the relationship between electrolytic resistance and mass water content of the respective concrete. It is determined by measuring the 2-electrode resistances on small specimens [58], when moisture equilibrium at a given relative humidity is reached. Hence, for young concrete the values of the mass water content can be compared only qualitatively. The measuring accuracy of the MRS is about 0.3% of water content [45].

Preliminary tensile and compressive tests were performed on specimens with and without embedded MRSs. The results confirmed that the sensor does not influence the measured longitudinal deformations. Moreover, even when the applied tensile load was increased until failure of the specimens, neither preferred failure in the vicinity of the sensor nor a significantly reduced uniaxial tensile strength was observed. Hence, the small reduction of the cross-sectional area due to the MRS was not taken into account.

## 3. Results and Discussion

### 3.1. Strain Evolution

**Figure 5:**
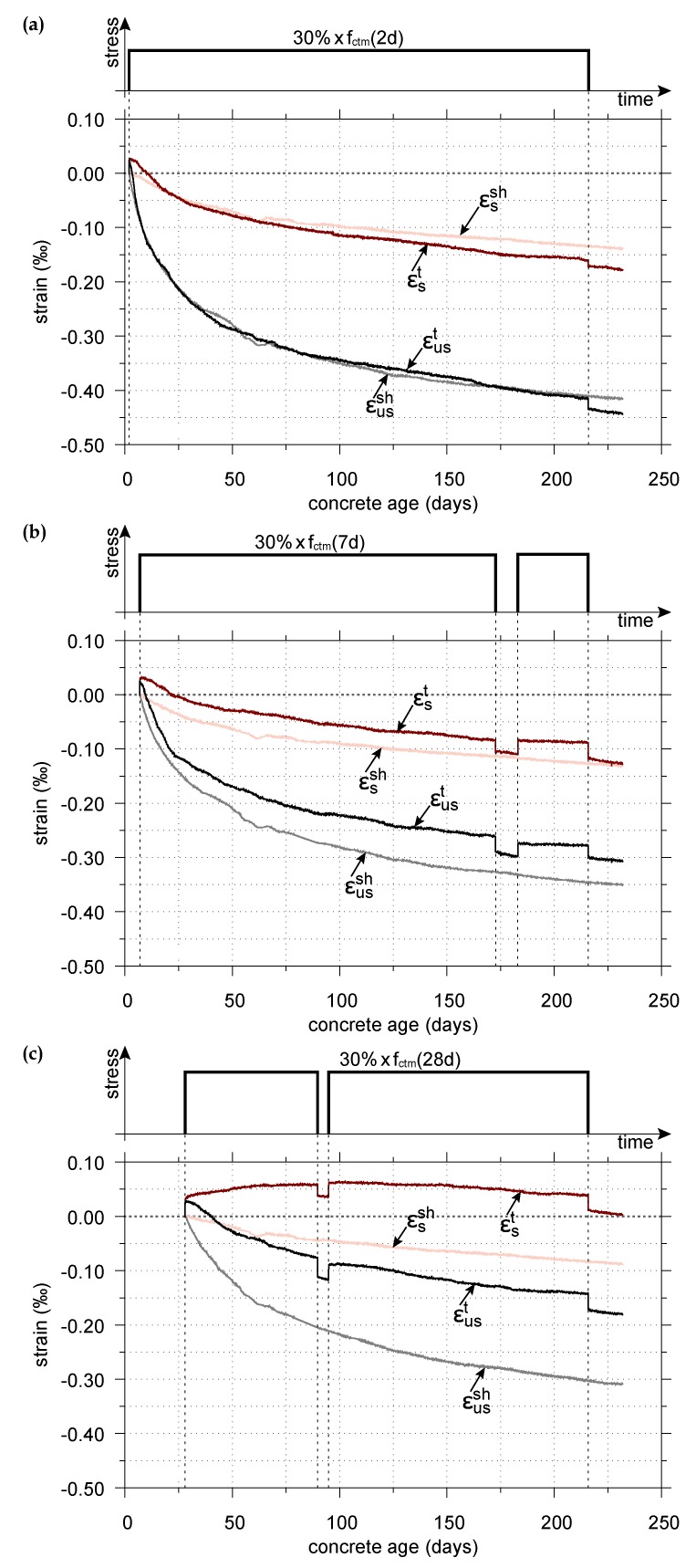
Evolution of the shrinkage strain and the total strain in sealed and unsealed specimens, both measured from the concrete age at loading of (**a**) $t_{0}$ = 2 days; (**b**) $t_{0}$ = 7 days; and (**c**) $t_{0}$ = 28 days to 30% of the average uniaxial tensile strength at the respective concrete age at loading.

Figure 5 depicts the measured evolution of the total strain $\varepsilon_{s}^{t}$ and $\varepsilon_{us}^{t}$, determined on sealed and unsealed specimens, respectively, loaded at the concrete ages of $t_{0}$ = 2, 7, and 28 days to 30% of the respective average uniaxial tensile strength at the concrete age at loading (schematically shown at the top of each figure), and the measured evolution of the respective autogenous shrinkage strain $\varepsilon_{s}^{sh}$ and combined autogenous and drying shrinkage strain $\varepsilon_{us}^{sh}$. The results of the shrinkage tests for both sealed and drying conditions have been recently published in [45] and are provided again in Figure 5 for completeness.

The total strain in sealed loaded specimens consists of the instantaneous elastic strain due to load application, the basic creep strain, and the autogenous shrinkage strain. The total strain in unsealed loaded specimens consists of the instantaneous elastic strain, the basic creep strain, the drying creep strain, and the combined autogenous and drying shrinkage strain.

For the sealed specimens, loaded at concrete ages of 2, 7, and 28 days, according to Figure 5, depending on the concrete age at loading the instantaneous total strain is followed by an increase of the total strain for a few hours, about one day and about 105 days, respectively. Subsequently, it decreases until unloading at the concrete age of 216 days. For the unsealed loaded specimens immediately after loading, the total strain decreases with progressing time.

This behavior can be explained as follows: The sealed and unsealed specimens of the creep tests are subjected to low tensile stresses corresponding to 30% of the average uniaxial tensile strength at the respective concrete age at loading, resulting in a positive instantaneous strain. In sealed specimens, the internal relative humidity of concrete decreases with progressing time due to cement hydration and the associated self-desiccation [59], resulting in internal uniform drying, whereas particularly in the surface zone of unsealed specimens, the decrease of the internal moisture content is the result of the interplay between self-desiccation and evaporation [59,60]. A decrease of moisture content results in an increase of the capillary pressure, acting on the solid skeleton of the concrete, which in turn results in negative strains [61]. Consequently, the instantaneous elastic strain and the increasing tensile creep strain are more than counteracted by the autogenous shrinkage strain or the combined autogenous and drying shrinkage strain, as also confirmed by other researchers [15,62]. Similar to the findings of Ji et al. [15], the results of the early age creep tests are characterized by a relatively large uncertainty since the total strain in the sealed and unsealed loaded specimens is of the same order of magnitude as the shrinkage strain in the respective load-free specimens.

However, with increasing concrete age at exposure to drying, the part of water chemically bound in the hydration products increases. Consequently, the capillary pressure acts on a stiffer solid skeleton [61], resulting in a smaller combined autogenous and drying shrinkage strain (cf. Figure 5). As recording of measurement data was started immediately after removing the sealing of the specimens, the portion of measured autogenous shrinkage is greater the earlier the specimen is exposed to drying. Similarly, the portion of measured autogenous shrinkage in the total strain determined on the sealed loaded specimens is greater the earlier the creep tests are started (cf. Figure 5). Hence, in both the sealed and unsealed loaded specimens, with increasing concrete age at loading, the respective measured shrinkage strain counteracts the instantaneous elastic strain and the tensile creep strain to a smaller extent. In addition, in the present study, the applied tensile stress is larger at higher concrete ages at loading. Thus, the difference between the measured total strain in the sealed loaded specimens and the measured autogenous shrinkage strain in the load-free companion specimen increases with increasing concrete age at loading. Similarly, the difference between the measured total strain in the unsealed loaded specimens and the measured combined autogenous and drying shrinkage strain in the load-free companion specimen increases with increasing concrete age at loading.

The creep recovery in tension is also shown in Figure 5 for the short term cycle of unloading and reloading of the specimens, loaded initially at the ages of 7 and 28 days, as well as after unloading all creep specimens at the concrete age of 216 days. The respective diagrams do not show any rapid recovery after removal of the tensile load and the recovery is nearly the same for both sealed and unsealed specimens (cf. Figure 5). These findings are consistent with the observations made by Illston [22].

Since it is common practice in the literature and in standards to express creep in terms of the compliance function, the respective compliance function for tensile creep will be determined subsequently. For this purpose, the autogenous shrinkage strain is subtracted from the total strain of the sealed loaded specimens, i.e., $\varepsilon_{s}^{t}-\varepsilon_{s}^{sh}$, whereas the combined autogenous and drying shrinkage strain is subtracted from the total strain of the unsealed loaded specimens, i.e., $\varepsilon_{us}^{t}-\varepsilon_{us}^{sh}$. The so-obtained load-induced strains are divided by the tensile stress applied in the respective creep test. This decomposition is based on the assumption of the additive composition of the strain components. Consequently, the combined autogenous and drying shrinkage strain in a loaded specimen is assumed to be equal to the one in a load-free specimen. Although controversially discussed [16,38,63,64], without the latter assumption, creep tests would have to be performed for all load levels planned in service, as explained in [63].

**Figure 6:**
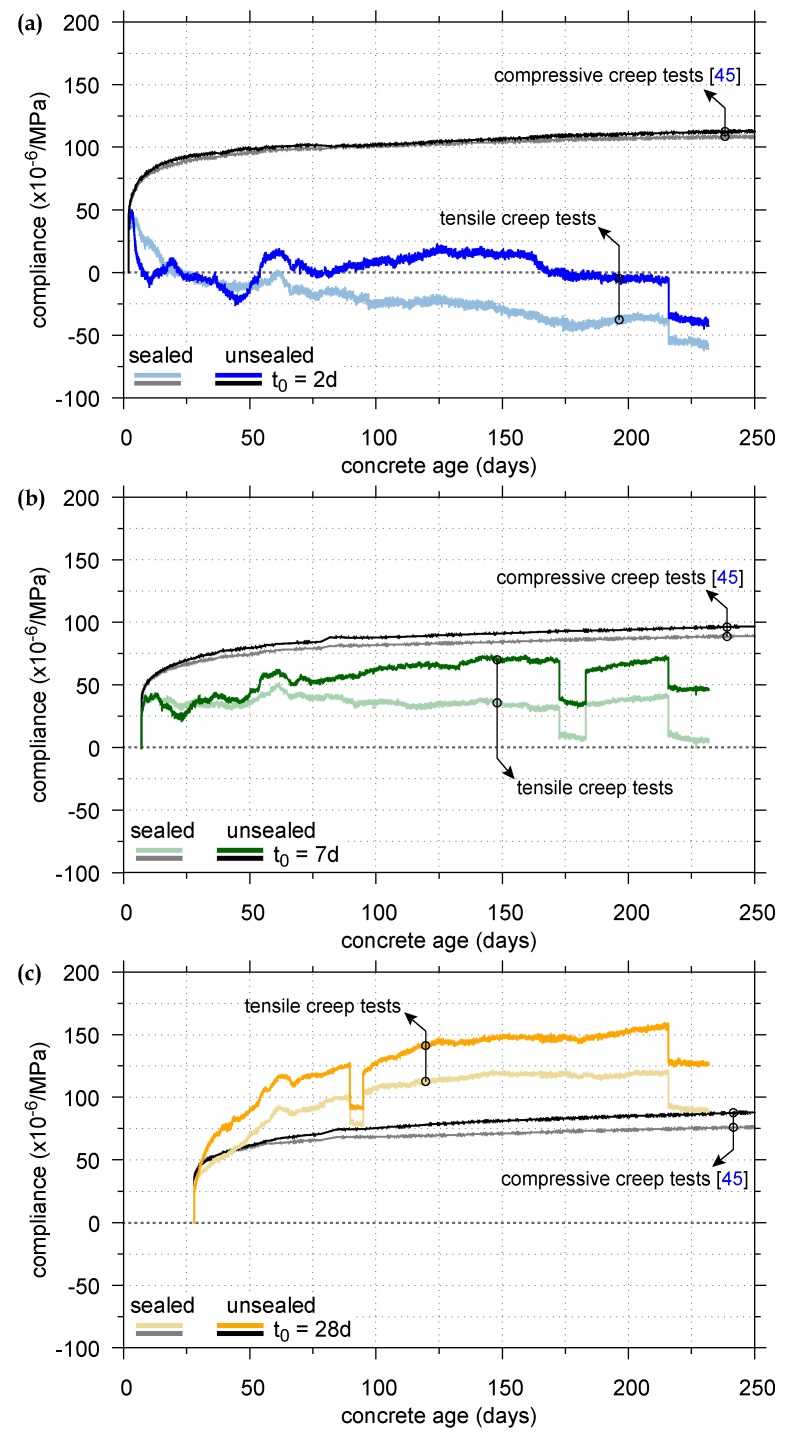
Compliance functions of sealed and unsealed specimens, loaded at the concrete ages of (**a**) $t_{0}$ = 2 days; (**b**) $t_{0}$ = 7 days; and (**c**) $t_{0}$ = 28 days to 30% of the respective average uniaxial tensile strength (colored curves) or 30% of the cylinder compressive strength (black curves).

Figure 6 shows the calculated compliance functions for the sealed and unsealed specimens, loaded at the concrete ages of $t_{0}$ = 2, 7, and 28 days to 30% of the respective average uniaxial tensile strength. Moreover, the results of creep recovery in tension after unloading the specimens are included. For comparison, the compliance functions obtained from the creep tests in compression [45] for the same concrete, the same load level, and the same concrete ages at loading, are shown. In contrast to Figure 6, the total strains, determined in the compressive creep tests, were not included in Figure 5, since they are of an order of magnitude larger than the total strains in tension.

The scatter of the compliance functions for creep in tension, depicted in Figure 6, is the consequence of calculating the differences $\varepsilon_{s}^{t}-\varepsilon_{s}^{sh}$ and $\varepsilon_{us}^{t}-\varepsilon_{us}^{sh}$ from very small measured strains of about the same magnitude. Despite those problems, the following trends can be observed: The evolution of the compliance of the sealed and unsealed specimens loaded in tension depends on the concrete age at loading and the test conditions. For example, the compliance of the sealed specimen, loaded at the age of two days, starts to decrease continuously about three days after loading, while the one of the unsealed specimen decreases quite sharply about 30 h after loading, and remains almost constant from the age of seven days until unloading at the age of 216 days with values close to zero. The compliance of the sealed specimen, loaded in tension at the age of seven days, remains almost constant until unloading, whereas the respective compliance of the unsealed specimen initially evolves quite similar to the one of the sealed specimen. However, at the age of about 50 days, the compliance of the unsealed specimen starts to increase slightly. The compliance of the sealed and unsealed specimens, loaded at the age of 28 days, is continuously increasing until unloading. Hence, for sealed and unsealed specimens loaded at the age of 28 days, the compliance increases due to an increase of the tensile creep strain. The latter is in line with the usual definition of the creep strain as the time-dependent increase of strain under sustained constant load [7]. On the contrary, for specimens loaded at the ages of seven days and two days, the compliance remains almost constant or even decreases, which is physically unreasonable. However, a review of the relevant literature shows that similar results were reported by researchers for basic tensile creep [13,15,16,17,26,27,64,65] and for drying tensile creep [66,67]. Several authors [16,17,26,27,64,65] concluded that negative or vanishing tensile creep strains are the consequence of the incorrect assumption of the additive decomposition of creep and shrinkage strain, i.e., the actual shrinkage strain of a loaded specimen is different from that of the load-free companion specimen. Hence, subtracting the autogenous shrinkage strain from the total strain of a sealed loaded specimen or the combined autogenous and drying shrinkage strain from the total strain of an unsealed specimen will not reflect the load-induced strain, as explained in [17].

Measuring of drying shrinkage strains at early concrete ages is associated with additional difficulties, as hydration and drying occur simultaneously and may interact [59]. Moreover, eigenstresses due to nonuniform drying arise and may induce additional microcracks at the specimen surface. These microcracks may relax parts of the eigenstresses (tensile stresses in the near-surface area and compressive stresses in the core) and decrease the potential for drying shrinkage [26,38]. In the case of tensile loading, more microcracks may be created on the surface of the unsealed loaded specimen compared to the load-free specimen [26], especially at early concrete ages at loading, when tensile strength is low. With progressing time, crack lengths and crack widths increase and, hence, the kinetics of drying shrinkage may be accelerated [26]. The latter is supported by the findings in [68,69], according to which cracking accelerates moisture diffusion in concrete.

Figure 6 shows that the compliance in tension under drying conditions is higher than the one under sealed conditions, as confirmed by other researchers [23,25,33]. At least for the sealed and unsealed specimens, loaded in tension at the age of 28 days, the compliance for tensile loading is greater than the compliance for compressive loading (cf. Figure 6), as confirmed by other researchers [21,22,23,24,25]. A comparison of the compliance functions for specimens loaded at the concrete ages of two and seven days cannot be made because of the physically questionable results of the respective compliance functions for creep in tension. For this reason, the Pickett effect for tensile and compressive creep is only evaluated for the concrete age of loading of 28 days: At the age of 216 days, the difference of the compliance in tension between sealed and unsealed specimens amounts to 32%, whereas it is only 15% for the compliance in compression. Hence, at least for the load level of 30% of the average uniaxial tensile strength or cylinder compressive strength, respectively, the Pickett effect in tension is more pronounced than in compression.

### 3.2. Moisture Content Evolution

**Figure 7:**
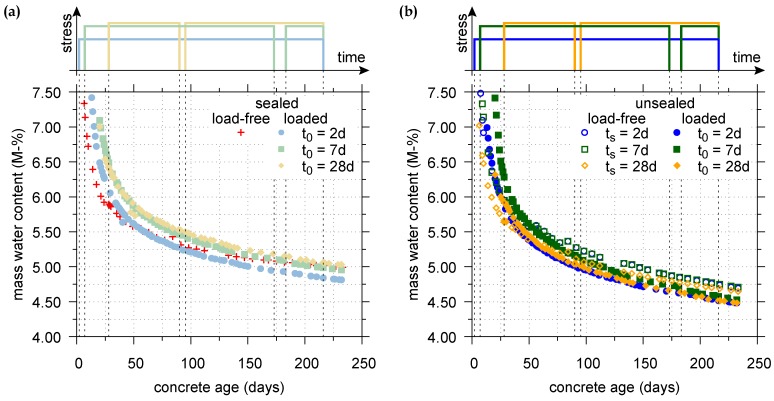
Evolution of the mass water content (**a**) for the sealed load-free specimen and sealed specimens, loaded in tension at $t_{0}$ = 2, 7, and 28 days; and (**b**) for unsealed load-free specimens and unsealed specimens, loaded in tension at $t_{0}$ = 2, 7, and 28 days, exposed to drying at the respective concrete ages $t_{s}$ or $t_{0}$, respectively.

Figure 7 displays the evolution of the mass water content for the sealed and unsealed specimens, loaded in tension at the concrete ages of $t_{0}$ = 2, 7, and 28 days. Moreover, the evolution of the mass water content for the sealed load-free specimen and for the load-free specimens, exposed to drying at the concrete ages of $t_{s}$ = 2, 7, and 28 days, recently published in [45], is shown for comparison. The depicted mass water content, measured by means of an MRS at the center of each specimen, represents the mean value of seven measuring points of a single MRS.

According to Figure 7a, the mass water content of the sealed load-free specimen decreases slightly slower than the ones of the sealed specimens loaded in tension. This finding is in accordance with the observations made by Reinhardt and Rinder [65]. Nevertheless, the differences are small and within the measuring accuracy of the MRS of about 0.3% of water content, indicating a similar evolution of the mass water content for the sealed load-free specimen and the sealed loaded specimens. Similar to the results obtained on sealed specimens, under drying conditions the mass water content of the load-free specimens decreases slightly slower than the ones of the specimens loaded in tension (cf. Figure 7b). However, again the differences are within the measuring accuracy of the MRS and, hence, under drying conditions, the evolution of the mass water content for the load-free specimens and the loaded specimens is also quite similar.

Furthermore, the short term cycle of unloading and reloading of the specimens does not show any effect on the evolution of the mass water content for both the sealed and unsealed loaded specimens (cf. Figure 7a,b).

## 4. Summary and Conclusions

The present contribution focused on the experimental investigation of the time-dependent behavior of a typical overlay concrete subjected to tensile stresses. The latter develop in concrete overlays due to shrinkage of the young overlay concrete, which is restrained by the substrate concrete. Since the tensile stresses are reduced by creep, creep in tension was investigated for sealed and drying conditions for different concrete ages at loading. Because of the dependence of shrinkage on the change of moisture content, in addition, the evolution of the mass water content was determined at the center of each specimen. Together with the experimental results for compressive creep from a previous study, a consistent set of time-dependent material data is now available. It has been determined for the same concrete mixture with the same shape and size of the specimens, the same sealing technique, the same measurement device, the same concrete age at loading and the same load level. Hence, any size effect due to different specimen sizes or any influence of different sealing techniques and measurement devices can be excluded. The set of measurement data consists of the autogenous shrinkage strain, the combined autogenous and drying shrinkage strain, the total strain, determined in tensile and compressive creep tests on sealed and unsealed specimens, loaded at concrete ages of 2, 7, and 28 days to 30% of the respective average uniaxial tensile strength or cylinder compressive strength, the respective compliance functions for creep in tension and compression and the evolution of the mass water content in sealed and unsealed specimens subjected to shrinkage and sustained tensile loading.

The following conclusions can be drawn from the present study:

- For early concrete ages at loading, the evolution of the total strain in specimens loaded to 30% of the respective tensile strength of concrete at loading is mainly governed by shrinkage due to hydration and drying. Consequently, the total strain in the sealed and unsealed loaded specimens and the respective shrinkage strain in the load-free companion specimens evolve quite similarly.
- For early concrete ages at loading, the compliance function indicates a vanishing or even negative tensile creep strain, which was also observed by other researchers. Hence, the usual assumption of the additive decomposition of creep and shrinkage strains is questionable, especially for early concrete ages at loading.
- At least for the concrete age at loading of 28 days, for both sealed and drying specimens, the compliance for tensile loading is higher than the one for compressive loading. Similarly, the Pickett effect is more pronounced in tension than in compression.
- Creep recovery in tension is nearly the same for both sealed and drying conditions.
- The evolution of the mass water content for both sealed and drying conditions does not show any noticeable impact of load-application on the moisture content and, in the long range the results for both sealed and drying specimens, loaded in tension, are within the measuring accuracy of the MRS.

The comprehensive set of material data for the investigated overlay concrete serves as valuable basis for calibrating models for the numerical simulation of the placement of concrete overlays. Appropriately calibrated numerical models will be used in parameter studies for optimizing the placement procedure of concrete overlays, starting from high-pressure water jetting of the substrate surface to the placement of the asphalt layers on the concrete overlay of the strengthened structure. Since the mechanical behavior of a concrete overlay is strongly influenced by additional physical phenomena, like thermal, hygral and chemical processes, a multiphase concrete model provides an appropriate description of the material behavior. In the latter, concrete is considered as a porous material, consisting of a solid skeleton and voids, filled by liquid water, dry air and water vapor. The multiphase model, available for this purpose, is based on the model proposed by Gawin et al. [61]. Recently, it has successfully been applied to model the material behavior of shotcrete [70] by considering interactions between the mechanical behavior and chemical, thermal and hygral processes, related to hydration and influenced by the ambient conditions.

## Figures and Tables

**Table 1:** Composition of the concrete mixture.

Components	Quantity	Unit
Cement CEM II A-M (S-L) 42.5N, *Lafarge*	375	$\mathrm{kg}/m^{3}$
Added water	165	$\mathrm{kg}/m^{3}$
Limestone sand 0/4 mm	810	$\mathrm{kg}/m^{3}$
Limestone aggregate 4/8 mm	183	$\mathrm{kg}/m^{3}$
Limestone aggregate 8/16 mm	457	$\mathrm{kg}/m^{3}$
Limestone aggregate 16/32 mm	367	$\mathrm{kg}/m^{3}$
Plasticizer *Proplast 200*	0.6	% ${}^{a}$
Air-entraining agent *Proair NVX*	0.045	% ${}^{a}$

${}^{a}$ percent of cement mass.

**Table 2:** Mechanical properties of the concrete determined on specimens sealed until testing.

Concrete Age	$f_{\mathrm{cm}}$	$E_{\mathrm{cm}}$	$f_{\mathrm{ctm},\mathrm{sp}}$	$f_{\mathrm{ctm}}$
(Days)	(MPa)	(MPa)	(MPa)	(MPa)
2	18.3 (0.2)	25,780 (660)	2.22 (0.06)	2.22 (0.12)
7	29.0 (2.0)	29,120 (370)	2.92 (0.09)	3.18 (0.06)
28	35.9 (0.7)	33,853 (630)	3.25 (0.29)	3.39 (0.05)

## Abbreviations

The following abbreviations are used in this manuscript:

*t*	concrete age, in days/hours
$t_{s}$	concrete age at exposure to drying, in days
$t_{0}$	concrete age at loading, in days
$f_{cm}$	average cylinder compressive strength, in MPa
$f_{ctm,sp}$	average splitting tensile strength, in MPa
$f_{ctm}$	average uniaxial tensile strength, in MPa
$E_{cm}$	average Young’s modulus, in MPa
$\varepsilon_{s}^{t}$	*t*otal strain in *s*ealed loaded specimens, in ‰
$\varepsilon_{us}^{t}$	*t*otal strain in *u*n*s*ealed loaded specimens, in ‰
$\varepsilon_{s}^{sh}$	autogenous *sh*rinkage strain in the *s*ealed load-free specimen, in ‰
$\varepsilon_{us}^{sh}$	combined autogenous and drying *sh*rinkage strain in *u*n*s*ealed load-free specimens, in ‰
LVDT	linear variable differential transformer
MRS	multi-ring-sensor
MP	measuring point
